# Disruption of ventricular activation by subthreshold delayed afterdepolarizations in RyR2-R420Q catecholaminergic polymorphic ventricular tachycardia^☆^

**DOI:** 10.1016/j.jmccpl.2025.100466

**Published:** 2025-06-11

**Authors:** Ewan D. Fowler, Salimata L. Diakite, Ana M. Gomez, Michael A. Colman

**Affiliations:** a School of Biosciences, College of Biomedical and Life Sciences, Cardiff University, Cardiff CF10 3AX, UK; b Inserm, UMR-S 1180, Signaling and Cardiovascular Pathophysiology, Université Paris-Saclay, 91400 Orsay, France; c School of Biomedical Sciences, Faculty of Biological Sciences, University of Leeds, LS2 9JT, UK

**Keywords:** Catecholaminergic polymorphic ventricular tachycardia, Optical mapping, Delayed afterdepolarizations, Arrhythmias, RyR2

## Abstract

**Background:**

Catecholaminergic polymorphic ventricular tachycardia (CPVT) carries increased risk of ventricular arrhythmias due to altered Ca^2+^ regulation associated with mutations in the ryanodine receptor (RyR2). Increased Ca^2+^ leak is believed to result in diastolic Ca^2+^ waves and delayed afterdepolarization (DADs) in cardiac myocytes, but it is uncertain how these cellular events induce ventricular tachycardia in the whole heart. We utilised a transgenic mouse model of human RyR2-R420Q (R420Q) CPVT mutation and a range of electrical and optical mapping technologies to examine the role of DAD-induced conduction abnormalities.

**Methods:**

Heterozygous R420Q and wildtype (WT) control hearts were perfused on a Langendorff apparatus. Electrical activity was monitored using volume conducted ECG electrodes and monophasic action potential (MAP) electrode recordings. Left ventricular activation and membrane potential changes were recorded using an 8 × 8 multielectrode array and optical mapping, respectively.

**Results:**

ECG recordings showed spontaneous ventricular arrhythmias in isolated R420Q hearts. More severe arrhythmias occurred in R420Q hearts following rapid electrical pacing combined with isoproterenol stimulation. Ventricular activation time was not different between genotypes, regardless of stimulation frequency or isoproterenol. Phase differences in local activation times were greater in R420Q hearts during 10 Hz pacing with isoproterenol, suggesting local conduction slowing. Optical mapping experiments revealed subthreshold DADs occurring in R420Q hearts during diastolic pauses. DADs prolonged the subsequent action potential and were associated with conduction slowing during the second beat after the DAD, but not the first beat. 2D tissue simulations revealed that direct inactivation of I_Na_ during DADs, or indirectly via cycle length dependent refractory mechanisms could account for local conduction slowing.

**Conclusions:**

Increased activation dispersion could arise from subthreshold DADs in R420Q mouse hearts and may contribute to conduction block. This could increase the propensity for re-entrant arrhythmias in CPVT without directly triggering ectopic beats.

## 1 Introduction

Catecholaminergic polymorphic ventricular tachycardia (CPVT) is a highly malignant arrhythmic heart disease, characterised by stress-induced ventricular tachycardia. Patients with the condition usually have structurally normal hearts and normal resting ECG but can develop serious ventricular tachyarrhythmias (VT), often brought on by physical or emotional stress [1]. CPVT is most commonly caused by autosomal dominant mutations in the ryanodine receptor (RyR2), or other proteins involved in excitation-contraction coupling.

Mutations that cause CPVT normally induce gain-of-function behaviour on RyR2 [2] resulting in excessive spontaneous Ca^2+^ release in cardiac myocytes in the form of Ca^2+^ sparks and Ca^2+^ waves that generate delayed afterdepolarizations (DADs) through the Na^+^/Ca^2+^ exchange (NCX) mechanism. If DADs exceed the threshold required for I_Na_ activation they can initiate focal triggered activity that can act as an ectopic pacemaker in the ventricle, but this requires the synchronisation of many cells to overcome the source-sink mismatch arising from electrotonic coupling of surrounding myocardium [3]. Subthreshold DADs that do not trigger ectopic action potentials but partially inactivate I_Na_ are also thought to be pro-arrhythmic by inducing local conduction slowing or unidirectional block and thereby generating a tissue substrate for re-entry [[4], [5], [6]].

Although subcellular Ca^2+^ handling defects are well established as a principal cause of CPVT, how these result in VT in the whole heart is less certain. Electrical or optical methods for mapping voltage and/or Ca^2+^ in the adult murine myocardium provide an opportunity to interrogate the link between subcellular defects and arrhythmogenesis in the intact organ [7]. Slower conduction velocity occurred in homozygous RyR2-P2328S mouse atrium and ventricle during pharmacological challenge with isoproterenol and/or caffeine [8,9], and this may have involved downregulation of Na_V_1.5 in this model [10]. In the absence of a change in effective refractory period, CV slowing will shorten the wavelength and increase the ability for re-entrant circuits to form.

Dysfunctional Ca^2+^ handling and DADs due to underlying CPVT mutations could impact the excitability or refractoriness of the myocardium [5,11]. To explore this further, we employed electrical and optical mapping technologies to investigate whether DAD induced conduction abnormalities could contribute towards the pro-arrhythmic phenotype in the heterozygous RyR2-R420Q murine model of clinical CPVT. The effects of this mutation on arrhythmias and cellular Ca^2+^ handling have been fairly well characterised in human patients [12], transgenic mice [[13], [14], [15]], and in human induced pluripotent stem cell-derived cardiomyocytes (hiPSC-CM) [16,17]. We found that subthreshold DADs may contribute to local conduction slowing in CPVT hearts during increased heart rate combined with catecholamine stimulation.

## 2 Methods

### 2.1 Transgenic RyR2-R420Q mouse model of CPVT

Experiments were carried out with local ethical approval and in accordance with the UK Home Office, Ministry of Research and Higher Education, and European Parliament Directive 2010/63/EU on the use of animals in research. Experiments were performed in adult male and female heterozygous RyR2-R420Q knock-in mice (R420Q) and wildtype littermates as controls (WT) on a C57BL/6J background. The generation of R420Q mice was previously described [13]. Mice were killed by stunning and cervical dislocation or under terminal anaesthesia via intraperitoneal injection of pentobarbital (100 mg/kg) with heparin (1000 IU/kg) in accordance with local procedures. Anaesthetic agents and heparin may influence cardiac function [18,19]. Hearts were removed and perfused on a Langendorff apparatus via the aorta with continuously oxygenated modified Tyrode's solution (containing, in mmol/L: 133 NaCl, 5 KCl, 1 NaH_2_PO_4_, 10 4-(2-hydroxyethyl)-1piperazineethanesulfonic acid (HEPES), 10 glucose, 1 or 1.8 CaCl_2_, 1 MgCl_2_), pH 7.4 with NaOH. Hearts were positioned horizontally in a temperature-controlled perfusion chamber (TBC-2.2, MappingLab, UK) maintained at 35 ± 1 °C.

### 2.2 Monophasic action potential recording

Monophasic action potentials (MAP) were recorded from the left ventricle apex using a twisted pair of Teflon coated chloridised Ag wires (0.25 mm in diameter) [20]. MAP signals were amplified with an isolated DC-coupled differential amplifier and lowpass filtered at 1 kHz. Amplified signals were digitized at 5 kHz using a Digidata 1440a (Molecular Devices, CA). Recordings were made in the presence or absence of isoproterenol (ISO, 100 nmol/L or 1 μmol/L). Blebbistatin (10 μmol/L) was included in the perfusion solution to minimise motion artefacts during MAP recording. Electrical stimuli were delivered via a pair of Ag electrodes using an isolated stimulus generator (SD9, Grass Instruments). Stimuli consisted of 2 ms monopolar pulses at constant voltage determined as 1.3× threshold required to entrain the heart to external pacing and, if necessary, the voltage was adjusted to maintain 1:1 capture over the course of the experiment. A stimulation protocol designed to elicit ventricular arrhythmias was performed that comprised a total of 21 stimulation sequences involving: a conditioning train of 20× S1 stimuli delivered at 10 Hz, followed by a burst of 10× S2 stimuli (varied between 17 and 50 Hz over the course of the protocol by incrementing the cycle length by 2 ms) [21], then pacing was switched off for 2 s to allow for detection of ventricular arrhythmias before then next sequence began. This protocol was applied to each heart and all data from the protocol were analysed for the severity of arrhythmia, if present.

Ventricular arrhythmias following burst pacing were classified as either single premature ventricular complexes (PVC), couplets, triplets, short VT (<1 s), or sustained VT (>1 s) according to published guidelines on the interpretation of preclinical arrhythmia research [22]. Bigeminy did not occur in any isolated hearts so this category was not used. A published scoring criterion was used to classify the severity of ventricular arrhythmia present [21], with scores for PVC = 1, couplet = 3, triplet = 4, VT(<1 s) = 5, VT(>1 s) = 6. A cumulative severity score was calculated for each heart based on the presence and type of ventricular arrhythmias that occurred in response to the burst pacing protocol. The cumulative score represented the sum severity of all arrhythmias that occurred during the stimulation protocol.

### 2.3 Multielectrode activation mapping

A pen-style 8 × 8 multielectrode array (MEA) with 0.42 mm interelectrode spacing (PA06408080101, MappingLab) was used to record ventricular activation sequence. Data were acquired at 10 kHz using a 64 channel digitizer (EMS64-USB-1003CS, MappingLab) and recorded using EMapRecord software. Activation time and conduction velocity were calculated in EMapScope software, with local activation time at each electrode being defined as the time of maximum negative deflection in the field potential, corresponding to the upstroke of the AP. The MEA was positioned on the left ventricle epicardium during pacing from the right ventricle. Local phase differences in activation times were calculated, and from this the 5th (P5) and 95th percentile (P95) of activation phase and the absolute dispersion of activation (P95-P5) were calculated. Absolute dispersion was normalised by the median dispersion (P50), to give the unitless activation dispersion index, (P95-P5)/P50, that accounts for possible differences in overall conduction [23]. MEA measurements were taken during steady pacing at the indicated frequency and the mean value from 3 to 10 consecutive beats was calculated for each heart.

### 2.4 Voltage optical mapping

Mouse hearts were removed and perfused as described above. A 1 mL bolus of the potentiometric membrane indicator, di-8-ANEPPS (50 μg/mL), was injected into a sideport of the Langendorff perfusion cannula over 5 min. Blebbistatin (10 μmol/L) was included in perfusion solutions during experiments to suppress motion artefacts. Excitation light was provided by four 532 nm LEDs passed through 530 ± 5 nm bandpass filters (FBH530-10, Thorlabs) that were positioned to give even illumination of the left ventricle. Fluorescence emission was collected through a 600 nm longpass filter (FELH0600, Thorlabs) and 12-bit image series were recorded at 900 Hz using a high sensitivity sCMOS camera (Kinetix22, Teledyne Photometrics). In some experiments, Ca^2+^ and V_m_ were monitored simultaneously by loading the heart with 25 μg Rhod-2-AM dissolved in 2 mL TYR with Pluronic-F127 (final concentration 0.2 %) over 10 min, and RH-237 (25 μg/mL in 1 mL) over 5 min. Excitation was as described above, but fluorescence was split using a Cairn OptoSplit with a 638 nm dichroic (DMLP638R, Thorlabs) and through additional 575 ± 25 bandpass (86-952, Edmund Optics) and 700 nm longpass filters (FELH0700, Thorlabs) to separate Rhod-2 and RH-237 signals, respectively, and directed onto different areas of the camera sensor. Image stacks were processed using a 3D Gaussian filter with FWHM of 250 μm in *x/y* and 1.3 ms in *t* to reduce high frequency noise, and further downsampled by binning to achieve a pixel dimension of 500 μm, similar to MEA spacing. Optical mapping data were analysed using the open-source ElectroMap software [24]. Pseudo-calibration of di-8-ANEPPS fluorescence was performed by scaling the normalised fluorescence to the typical resting (−80 to −70 mV) and peak V_m_ (+20 to +30 mV) reported *in situ* in mouse ventricle [20], to give an approximation of V_m_ during optical mapping experiments.

### 2.5 Computational modelling

Simulations were performed using the Multiscale Cardiac Simulation Framework [25,26], which integrates cellular and tissue models with functionality for imposed and controlled spontaneous calcium release functions to represent the dynamics and stochastic nature of calcium induced DADs. Cellular electrophysiology was modelled using a modified version of our previously presented rat ventricular model [27]. The reversal potential of *I*_K1_ was shifted by 15 mV compared to the original model, to give a resting membrane potential of approximately -70 mV, typical of mouse ventricle [20]. This feature was important to reproduce due to its large impact on the dynamics and impact of DADs. The ionic model was then integrated with the non-spatial approximation of the intracellular calcium handling model originally presented, making it suitable for integration with the reduced model of spontaneous activity.

Spontaneous calcium release events (SCRE) that underlie DADs were imposed using the spontaneous release function approach previously presented and implemented in various studies [6,26,28]. This enables independent and direct control over the distributions that determine the timing and magnitude of SCRE and, therefore, different conditions can be explored in a wide parameter space. Hence, the direct impact of CPVT mutations on the vulnerability to SCRE were not included, as it is the SCRE dynamics themselves that become the control variable.

Tissue models were constructed of different sizes, comprising a 2D sheet of either 100 × 100 nodes or 300 × 300 nodes at a spatial discretisation, Δx, of 0.15 mm (i.e., full tissue dimensions of 15 × 15 mm^2^ or 45 × 45 mm^2^, significantly larger than the area mapped through the MEA). Myocyte orientation was varied smoothly throughout this space as in a previous study [28] and diffusion parameters were set to D1 = 0.4 mm^2^/ms with an anisotropy ratio of 7, resulting in conduction velocities of 0.792 m/s and 0.298 m/s, longitudinal and transverse to the fibre direction, respectively. Cellular coupling was modelled using the well-established anisotropic finite differences method. Different locations were selected as stimulus sites and simulations were conducted across the range of relevant parameters. To compare to experimental data, a smaller region within the tissue model corresponding to the extent of the mapping arrays was extracted and the data were down-sampled by a factor of three to reproduce approximately the spacing of MEA electrodes.

All tissue models were paced under normal conditions at both 10 Hz and 6.6 Hz to steady-state, and the state variables saved. These were then read in for the start of the results simulations, wherein one or multiple further stimuli were applied while also imposing the spontaneous release functions, and the resulting activation sequence analysed.

### 2.6 Statistics

Data were checked for normality using Shapiro-Wilk test. Data with a normal or log-normal distribution were analysed using parametric statistics, otherwise non-parametric alternatives were used. Data are presented as mean ± SEM. *P* < 0.05 was considered statistically significant.

## 3 Results

### 3.1 ECG measurements in perfused R420Q hearts

**Fig. 1:**
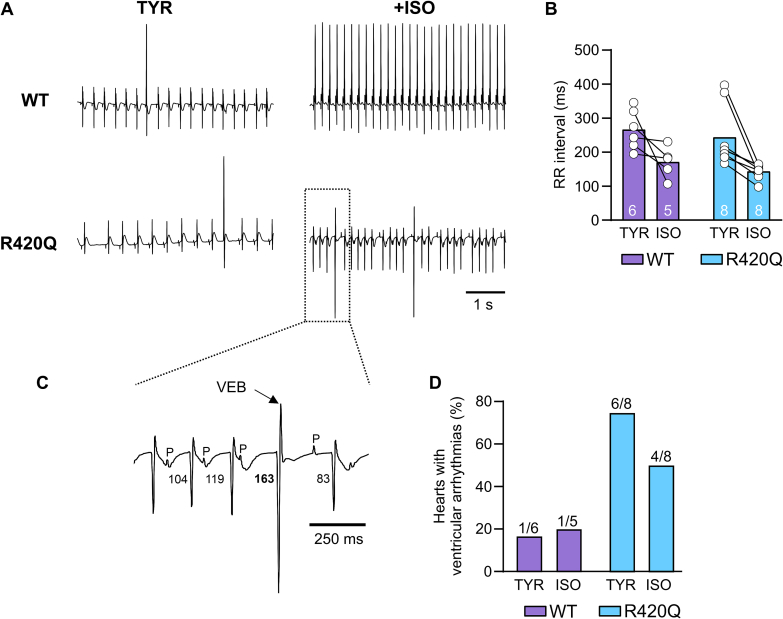
Spontaneous ventricular arrhythmias in perfused R420Q hearts under basal conditions (1.8 mM Ca^2+^ Tyrode, TYR) and with catecholamine stimulation (1 μmol/L isoproterenol, ISO). A Exemplar sinus rhythm ECG recordings in WT and R420Q hearts with and without ISO stimulation. B RR interval in perfused hearts under basal or ISO stimulation. C Expanded timescale view of the outlined area in (A) showing a PVC occurring in an R420Q heart during ISO stimulation. The PQ interval is prolonged just prior to the PVC, suggesting possible delayed AV conduction compared to surrounding beats (PQ intervals are indicated on the figure in ms). D Number of hearts that developed spontaneous ventricular arrhythmias. The number of hearts used is indicated on the bars in (B&D). One WT heart did not have data in ISO.

The RyR2-R420Q CPVT mouse model has previously been shown to develop similar stress-induced arrhythmias *in vivo* as patients with the same mutation, such as bidirectional VT, sinus bradycardia, and junctional escape [13,14]. Fig. 1A shows exemplar ECG recordings from explanted Langendorff perfused WT and R420Q hearts that were devoid of autonomic control. The RR interval was calculated during a 5 s recording and was similar between WT and R420Q hearts in basal conditions with 1.8 mmol/L extracellular Ca^2+^, and also during ISO stimulation (1 μmol/L) (Fig. 1B). Fig. 1C shows a ventricular ectopic beat (VEB) occurring during the R420Q recording in Fig. 1A on an expanded time scale. Spontaneous ventricular arrhythmias, such as ventricular tachycardia, VEB and intermittent pauses, occurred in 6/8 R420Q hearts under basal conditions and in 4/8 hearts under ISO stimulation, whereas these occurred in only 1/5 WT hearts (Fig. 1D). The slight decrease in R420Q hearts exhibiting arrhythmias in response to ISO under these conditions could indicate that Ca^2+^ cycling was already near-maximal in R420Q hearts with 1.8 mmol/L external [Ca^2+^], or that the positive chronotropic response to ISO suppressed some forms of arrhythmia [29]. We conducted further experiments under less potentiated conditions (1 mmol/L Ca^2+^ ± 100 nmol/L ISO) and found that the incidence of spontaneous arrhythmias was similar in WT (1/10 hearts) and R420Q (2/11 hearts) in TYR, whereas in 100 nmol/L ISO 5/11 R420Q hearts developed arrhythmias compared to 0/10 WT hearts (Fig. S2). To investigate changes in response to adrenergic stimulation, subsequent experiments used 1 mmol/L Ca^2+^ ± 100 nmol/L ISO, unless stated otherwise.

### 3.2 Rapid pacing induced ventricular arrhythmias in R420Q hearts

**Fig. 2:**
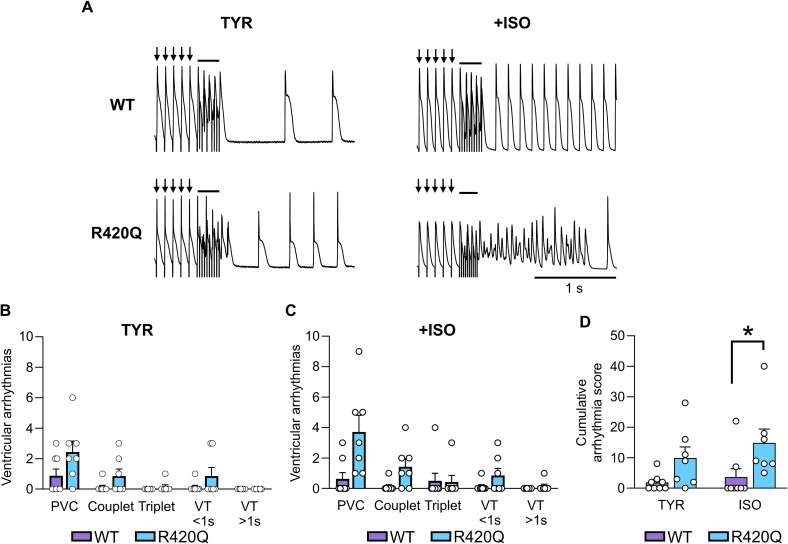
Increased arrhythmia susceptibility to rapid pacing in R420Q hearts during beta-adrenergic stimulation with 100 nmol/L isoproterenol (ISO) in 1 mmol/L Ca^2+^ Tyrode's (TYR). A Monophasic action potential recordings in WT and R420Q hearts during a single sequence of electrical pacing at 10 Hz (arrows) with a short burst of rapid pacing (solid line) followed by no electrical pacing in TYR (left panels) and then in ISO (right panels). This pacing sequence was repeated a total of 21 times with the burst frequency varied between 17 and 50 Hz. B Number and type of ventricular arrhythmia elicited by rapid pacing in TYR and C in ISO. D The types of ventricular arrhythmia were scored by severity and duration and the cumulative arrhythmia score for the burst pacing protocol was calculated. Symbols in B–D represent counts from each heart. (B–D) *N* = 8 WT and 7 R420Q hearts. (D) One-way ANOVA on ranks. *P < 0.05.

To investigate these arrhythmias further, monophasic action potentials (MAP) were recorded in perfused hearts from WT and R420Q. A sequence of programmed electrical stimulation was applied as a conditioning train of S1 stimuli at 10 Hz followed by a burst of 10 S2 stimuli between 17 and 50 Hz, then pacing was switched off to allow for detection of ventricular arrhythmias (Fig. 2A). One or more triggered beats occurred after S2 pacing was stopped in 4/8 WT and 6/7 R420Q hearts in TYR. The types of arrhythmias were categorised following guidance on arrhythmia classification in preclinical models [22]. Single premature ventricular complexes (PVC) and couplets were the most common events in WT and R420Q hearts in TYR (Fig. 2B). Ventricular arrhythmias occurred in 2/8 WT hearts during ISO stimulation, whereas they occurred in all (7/7) R420Q hearts under the same conditions (Fig. 2C). In several R420Q hearts these included more severe forms such as short VT (<1 s) and in one case longer lasting VT > 1 s (this heart is shown in the lower panels in Fig. 2A). The ventricular arrhythmia burden was scored for cumulative severity based on published criteria [21]. The cumulative severity score was not different between genotypes in TYR, whereas the severity was greater in R420Q hearts compared to WT in ISO (Fig. 2D).

### 3.3 Total activation time was not different in R420Q hearts

**Fig. 3:**
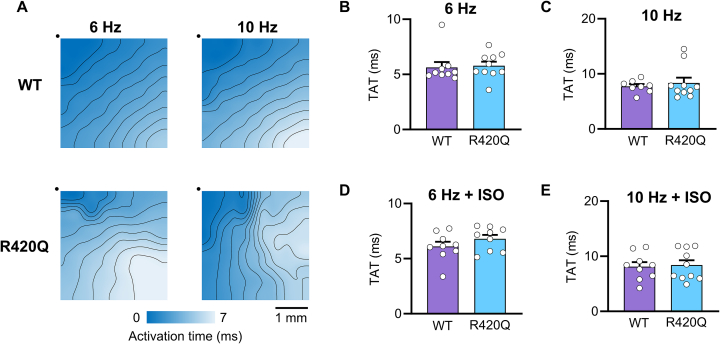
Total activation time (TAT) was not different between WT and R420Q hearts in 1 mmol/L Ca^2+^ Tyrode's (TYR) with or without 100 nmol/L isoproterenol (ISO). A Ventricular epicardial activation maps generated from multielectrode recordings in a WT and R420Q heart during electrical pacing at 6 or 10 Hz in TYR. Black dots indicate the location of MEA electrode #1 and the site closest to stimulating electrode. B–E TAT was not different between WT or R420Q hearts in either TYR or ISO at any pacing frequency. N = 9 WT & 10 R420Q hearts (B,C) Unpaired *t*-test, (D,E) Mann-Whitney test.

We next investigated whether conduction slowing might contribute towards increased vulnerability to arrhythmias. We performed electrical mapping of the left ventricle epicardium in perfused hearts using a pen-style 64-channel multielectrode array during pacing from the right ventricle. Fig. 3A shows exemplar maps of activation time in a WT and R420Q heart during steady pacing at 6 and 10 Hz. Total activation time (TAT) was not different between WT and R420Q hearts at either 6 Hz or 10 Hz in TYR (Fig. 3B,C). As expected, there was a rate-dependent increase in TAT in WT and R420Q hearts (*P* < 0.001, two-way repeated measures ANOVA) but this was not different between genotypes. Similarly, TAT was not different between WT and R420Q hearts during ISO stimulation at either 6 or 10 Hz (Fig. 3D,E).

The average of local conduction velocity (CV) vectors was calculated and these data are presented in Supplementary Table 1. Similar to TAT, there were no differences in CV between WT and R420Q hearts at either 6 or 10 Hz, in the presence or absence of ISO. This was somewhat surprising, given that there appeared to be some narrowing of isochrones in R420Q hearts during faster pacing (e.g. lower panels in Fig. 3A). This was not due to underlying structural differences because the stimulating and recording sites were the same for both pacing protocols. This is also evident in individual electrograms covering the region of delayed activation taken at the onset and later during pacing (Fig. S3). The pathway an electrical impulse follows through the 3D myocardium during epicardial pacing will be affected by tissue anisotropy, for instance resulting from fibre orientation, and potentially by retrograde activation of the fast conduction system that can result in breakthroughs distal to the pacing site. Since the exact pathway is unknown this could over- or underestimate absolute conduction values. We followed the approach of Lammers *et al.* [23] and calculated local phase differences in activation to detect areas of inhomogeneity, which does not require the direction of propagation to be known.

### 3.4 Local delays in tissue activation increase activation dispersion

**Fig. 4:**
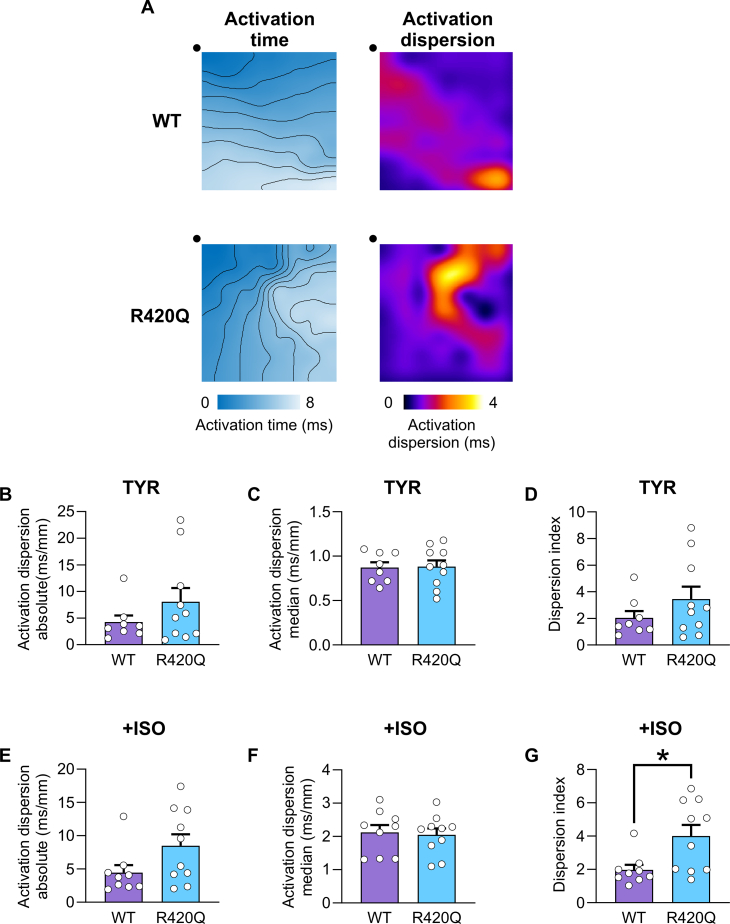
Activation dispersion is increased in R420Q hearts during 10 Hz pacing in 1 mmol/L Ca^2+^ with beta-adrenergic stimulation (100 nmol/L ISO). A Ventricular activation maps in WT and R420Q hearts during 10 Hz stimulation in ISO (left panels). Local activation phase maps were calculated (right panels) and show more clearly local delays in activation occurring in R420Q. B Absolute activation dispersion (P95-P5), C median activation dispersion (P50) and D dispersion index ([P95-P5]/P50) were not different between WT and R420Q during 10 Hz pacing in TYR. E There was a slight, although not significant (*P* = 0.076), increase in absolute dispersion in ISO in R420Q hearts, F without a change in median dispersion, G which resulted in an increase in activation dispersion index. N = 9 WT & 10 R420Q hearts. (B–G) unpaired *t*-test. *P < 0.05.

Fig. 4A shows exemplar activation time maps during 10 Hz pacing with ISO stimulation. The activation pattern is fairly uniform in the WT heart, whereas in the R420Q heart there is a region where the isochrones are more compact, indicating local activation delays. This is more obvious in the corresponding phase maps in Fig. 4A, calculated as the maximum absolute difference in local activation time, that were used to calculate local activation dispersion. Absolute and median activation dispersion, and dispersion index, were not different between WT and R420Q hearts in TYR paced at 10 Hz (Fig. 4B–D). ISO increased the absolute dispersion, although not significantly, without altering the median dispersion (Fig. 4E,F). The activation dispersion index, which corrects for the median activation dispersion, was greater in R420Q than in WT hearts at 10 Hz in ISO (Fig. 4G), indicating that some local conduction slowing may be occurring. MEA is an extracellular mapping technique and cannot directly identify the cause of conduction slowing, so in further experiments we employed voltage optical mapping of the left ventricle epicardium.

### 3.5 Subthreshold delayed afterdepolarizations (DAD) during voltage optical mapping

**Fig. 5:**
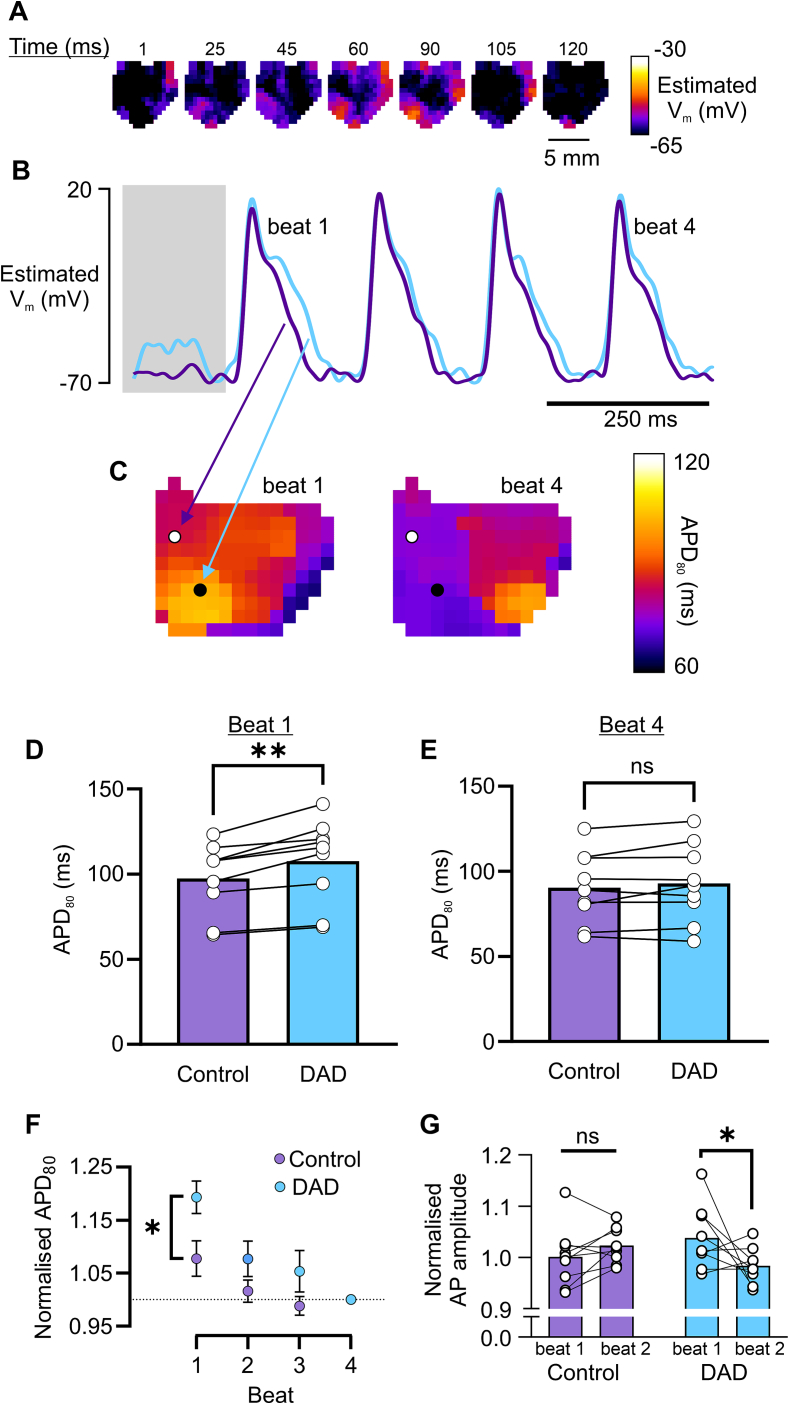
Delayed afterdepolarizations prolong action potential repolarization and influence local conduction slowing in R420Q hearts imaged using optical voltage mapping in 1 mmol/L Ca^2+^ and 100 nmol/L ISO. A Pseudo-calibrated voltage maps of an R420Q heart at different timepoints during a prolonged diastolic pause following pacing at 10 Hz (indicated by the shaded box in B). A localised subthreshold depolarization is visible in the bottom left of the maps. B Estimated V_m_ at two different regions where a DAD was absent (purple line) or present (blue line). C Maps of APD_80_ during the 1st and 4th beat in (B). D APD_80_ was greater in DAD regions compared to control regions during beat 1, whereas E APD_80_ was not different between DAD and control regions by beat 4. F APD_80_ values normalised by the near steady-state value (beat 4) G AP amplitude (normalised to beat 4) was not different in control regions between beat 1 and 2, whereas the amplitude decreased in DAD regions in beat 2, which could be indicative of slower conduction. (D–G) N = 9 R420Q hearts. (D,E) paired t-test, (F,G) two-way repeated measures ANOVA. (For interpretation of the references to colour in this figure legend, the reader is referred to the web version of this article.)

DADs are thought to be the underlying cause of tachyarrhythmias in CPVT, although exactly how these initiate ectopic beats in the whole heart is less understood. We hypothesised that DADs may underlie local activation slowing in R420Q hearts, that could give rise to unidirectional conduction block. To increase the likelihood of DADs occurring, R420Q hearts were challenged with ISO and paced at 10 Hz, then pacing was switched off to allow a relatively longer diastolic pause before sinus rhythm resumed. An example of one of these events is shown in the voltage maps in Fig. 5A, in which a localised low amplitude depolarization occurs near the apex at the bottom of the maps. Diastolic depolarizations occurred in localised regions of the ventricle in 9/10 R420Q hearts, but these did not directly initiate ventricular ectopic beats. The exemplar recording in Fig. 5B shows the voltage profile in the region with diastolic depolarization and at a remote region. The mean duration of these events was 115 ± 9 ms. Although the absolute membrane potential cannot be determined from these recordings, if we assume a typical resting potential in the range of −80 to −70 mV, and AP peak around +20 to +30 mV, as reported in mouse ventricle [20], then the fairly linear voltage response of di-ANEPPS dyes [30] does allow pseudo-calibration of optical signals. Using this approach, we estimate the peak voltage of these events to be in the range of approximately −58.4 ± 1.6 mV to −52.4 ± 1.3 mV, which would be consistent with subthreshold DADs, although it should be made clear these values are only illustrative.

### 3.6 Impact of DADs on action potential duration and conduction in R420Q hearts

The exemplar APD_80_ maps in Fig. 5C correspond to the 1st sinus beat and the near steady-state (4th beat) from the same recording as Fig. 5A, with the location of the DAD indicated by a black circle and a nearby region without a DAD that served as an internal control (white circle). The APD of the first beat was much greater than the 4th beat in the DAD region (102 vs 72 ms), whereas in the control region the difference in APD between 1st and 4th beats was much smaller (84 vs 74 ms). In the 9 R420Q hearts in which DADs were identified, the first APD following a DAD was longer in that same region than in control regions (Fig. 5D), suggesting they could be causally linked. Alternatively, it could be argued that regions with DADs may be in some way damaged, or intrinsically different to control regions, and therefore more prone to both DADs and AP lengthening. However, there was no difference in the near-steady state APD between different regions (Fig. 5E), showing that this was not due to inherent regional differences in APD or tissue viability, but likely occurred because of the DAD itself. This relationship was maintained when APD values were expressed relative to the steady state APD in the same region (Fig. 5F). It is likely that SR depletion and consequently less Ca^2+^-dependent inactivation of I_Ca_ contributed to AP lengthening during the first beat following a DAD [11]. In additional experiments, simultaneous Ca^2+^/V_m_ mapping was employed using Rhod-2 and RH-237 loaded hearts.

SCRE were detected during the pause following 10 Hz pacing in 3/3 R420Q hearts. Fig. S5 shows an exemplar recording from an R420Q heart in which SCRE slightly reduced the Ca^2+^ transient amplitude by ∼3 % and prolonged its duration, compared to the steady-state Ca^2+^ transient properties in the same region, as might be expected if the SR was unloaded. In contrast, in different recordings from the same heart and region of tissue, but where diastolic Ca^2+^ release did not occur, there was a slight increase in amplitude of the first post-pause Ca^2+^ transient compared to steady state (also by ∼3 %), consistent with greater diastolic SR refilling. The APD of the first beat was ∼6 % longer when SCRE occurred compared to without, which was consistent with the previous findings using di-8-ANEPPS, albeit with SCRE having a slightly smaller effect on APD. Simultaneous voltage DADs corresponding to SCRE were not clearly discernible, suggesting that the additional cytosolic Ca^2+^ buffering by Rhod-2 might reduce activation of NCX and impact on V_m_ [31]. For these reasons, no further experiments to record Ca^2+^ were performed.

In di-8-ANEPPS loaded hearts, the longer APD of the first beat shortened the diastolic interval between first and second beats slightly to 138.1 ± 19.5 ms in regions with DADs, compared to 148.4 ± 21.2 ms without DADs (*P* < 0.01; paired *t*-test). DADs can increase I_Na_ inactivation and slow conduction [5]. To see whether DAD regions were associated with local conduction slowing, we compared the amplitude of APs in regions with and without DADs. We reasoned that spatial averaging will reduce the optical AP amplitude, and that this will become more apparent as conduction slows due to some parts of the tissue encompassing a pixel having yet to activate. However, there was no difference in AP amplitude during the first beat following a DAD, compared to regions without a DAD, suggesting that subthreshold DADs did not significantly slow local conduction during the first beat (Fig. 5G). However, the amplitude of the second AP following a DAD was reduced compared to the previous beat, which did not occur in the control regions. We also used the maximum rate of rise of the optical AP (dF/dt_max_) as an indicator of local conduction [32]. Fig. S4 shows the relationship between dF/dt_max_ of the first and second beat after a DAD, plotted as a function of the interval between the DAD and the first beat. There was a significant positive correlation between DAD interval and dF/dt_max_ of the second beat in DAD regions (*P* = 0.012), but not in the first beat or in corresponding control regions during either beat. A possible explanation for this could be the shorter diastolic interval in DAD regions causing incomplete recovery from inactivation of I_Na_. We performed computer modelling of 2D cardiac tissue to separate the functional consequences of direct I_Na_ inactivation during a DAD from I_Na_ inactivation during prolonged APs and assess whether either of these possibilities could account for local conduction slowing in R420Q hearts.

### 3.7 Computational modelling

**Fig. 6:**
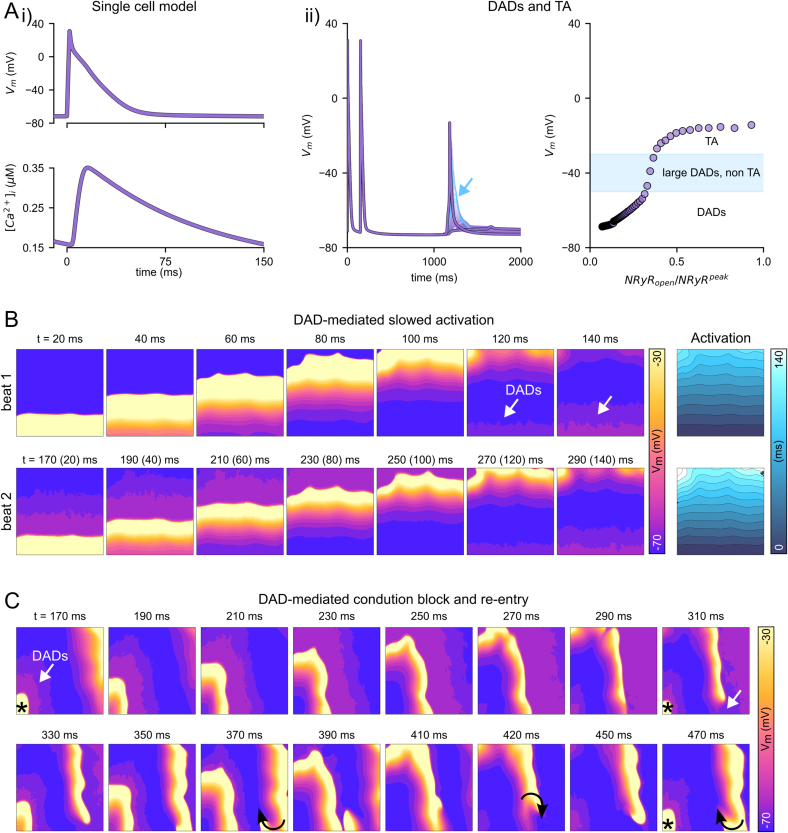
Simulation of DAD mechanisms of conduction block. Ai Illustration of the action potential (upper) and calcium transient (lower) of the modified integrated cell model. Aii Impact of SCRE of varying magnitude (N_RyR_open_/N_RyR_^peak^) on DADs and the action potential in single cell. Blue indicates the large but non-TA inducing DADs that occur due to *I*_Na_ inactivation. B Temporal voltage snapshots and the resulting activation sequence of two sequential paced excitations, with DADs occurring in advance of the second excitation. Note that the voltage colour map has been scaled to a maximum of −30 mV, and has been discretised into 5 mV contours, as these settings best illustrate the DADs, which can be challenging to see if the voltage is scaled to the true peak at above 0 mV. C Temporal snapshots of another simulation in which localised conduction block as a consequence of the DADs occurred and developed into a sustained re-entrant pattern (only on the larger 300 × 300 tissue model). The voltage colour scale is the same as that in panel B. (For interpretation of the references to colour in this figure legend, the reader is referred to the web version of this article.)

Single cell models were modified to better fit our murine experimental model (see Section 2.5). These reproduced some of the key features of mouse electrophysiology, including an APD_90_ 51.3 ms and a resting membrane potential of −70.4 mV (Fig. 6Ai). Imposing spontaneous Ca^2+^ release functions with a varying magnitude illustrates a gradually increasing magnitude of DADs, eliciting full triggered activity (TA) above a certain threshold (approximately −50 mV) (Fig. 6Aii). Large but non-TA inducing DADs were also observed within an SCRE magnitude range, which is primarily a consequence of the relatively depolarised resting membrane potential [6,28]. APD had a non-linear dependence on DAD properties, largely due to the magnitude of the resulting Ca^2+^ transient having opposing effects of reduced I_CaL_ inactivation (prolonging APD) versus reduced I_NCX_ (shortening APD) (Fig. S8).

The first set of tissue simulations was performed by imposing SCRE distributed globally throughout the tissue, with each individual node of the tissue model undergoing SCRE defined by the statistics of the input parameters (i.e., varying the synchronisation degree, the mean magnitude, and the variation of magnitude throughout the tissue). The specific values of the timing and magnitude for each node were randomly sampled from these given distributions on a beat-by-beat basis. These global simulations revealed that DADs that precede an excitation can slow down the propagation velocity throughout the tissue (Fig. 6B). However, no parameters were found wherein localised conduction failure was observed without the context of this global reduction to conduction velocity: either SCRE was insufficient to lead to any impact, or the impact was global. Within this context of global conduction slowing, further localised failure and conduction block could be observed, which could lead to re-entrant dynamics in the largest tissue model (Fig. 6C). This global conduction slowing was not observed in the experiments, indicating that spontaneous activity may be localised to specific regions of the tissue. Therefore, to explore whether DADs can explain the experimental observations, further simulations were performed in which SCRE was constrained to local patches of varying size.

### 3.8 Interactions between DADs and subsequent APDs contribute to local conduction slowing

**Fig. 7:**
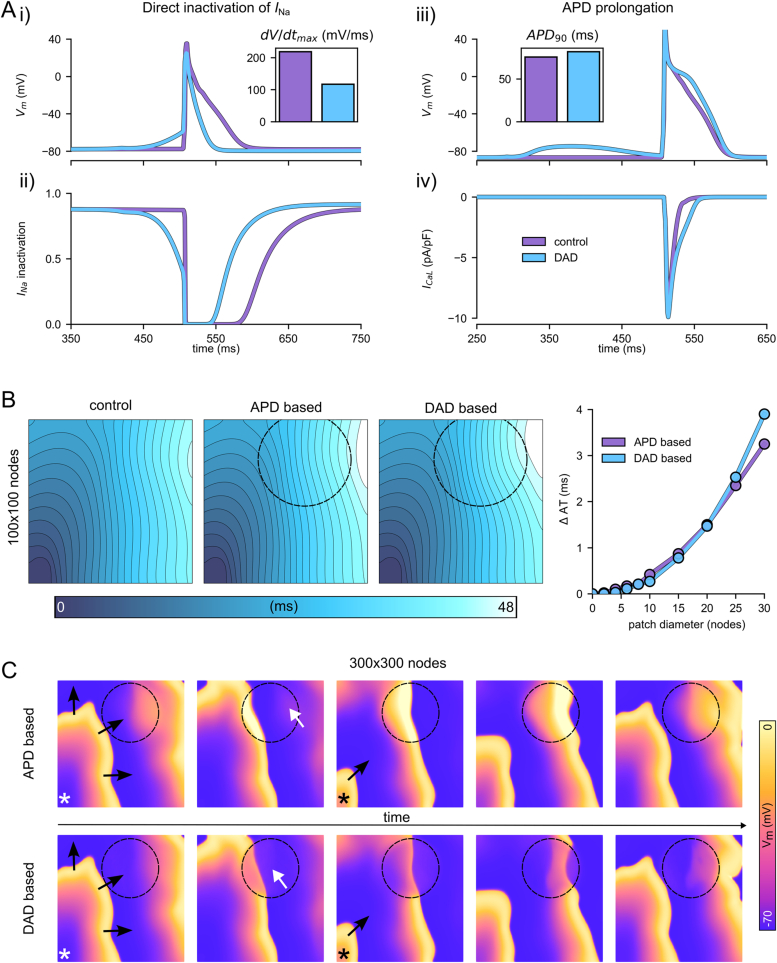
Simulation of different mechanisms of localised conduction block. A Illustrative single cell simulations of the two different mechanisms by which DADs could result in conduction block. *I*_Na_ inactivation (Aii) is given by the combination of the two voltage-based inactivation gates. B Localised conduction slowing in the two different mechanisms, with illustrated activation maps corresponding to a patch size of 30 nodes (indicated by the circle), and the summary of the difference in latest activation time compared to control versus patch size. C Voltage snapshots in the larger tissue model that illustrate the mechanism of localised conduction slowing. Black arrows indicate wavefront propagation; white arrows highlight the prolonged APD/DADs that interrupt the next excitation.

These simulations aimed to explore the differing impact of two independent mechanisms by which DADs can result in conduction block: 1) direct inactivation of *I*_Na_ through the increased membrane potential associated with the DAD and 2) a prolonged APD of the subsequent beat as a consequence of reduced *I*_CaL_ inactivation due to SR depletion. Both of these mechanisms are shown (for illustrative purposes) in Fig. 7A. It is worth noting that these two mechanisms have some important distinct features: Firstly, the timing of the stimulated AP relative to the DAD is required to occur during the DAD for the direct *I*_Na_ inactivation mechanism, which is voltage dependent, whereas the APD prolongation mechanism more readily occurs when the AP is timed during the tail or after the DAD, due to the requirement for calcium unloading to inhibit CICR. Secondly, the timing of the conduction failure relative to the DAD would also be different for these mechanisms, occurring on the beat immediately following the DAD for the direct *I*_Na_ inactivation mechanism, and on the subsequent beat for the APD mechanism (i.e., following the APD prolongation induced by the DAD).

In order to fully separate the two mechanisms, APD prolongation was tested by directly prolonging the APD in the localised region, through an increase in *I*_CaL_ by a factor of two and reduction to *I*_to_ by a factor of 0.75_,_ prolonging APD_90_ by 12 ms to 63.4 ms, rather than being a consequence of the DADs. Therefore, we will refer to them as the “APD-based” and “DAD-based” mechanisms for clarity, though it should be noted that in the experiment the APD prolongation is likely a consequence of the DADs and not of local heterogeneities in *I*_CaL_ and *I*_to_. At a pacing frequency of 10 Hz the APD_90_ was 50 ms and the dV/dt_max_ was 75 mV/ms in control conditions; following APD prolongation on a single AP (i.e., imposed once at the end of the steady-state train of stimuli), the APD_90_ was 73 ms and the dV/dt_max_ of the subsequent excitation was 55 mV/ms. The generally low dV/dt_max_ values are a consequence of the relatively depolarised resting potential at around −70 mV.

Imposing DADs or APD prolongation in localised patches of varying sizes demonstrated that both mechanisms can result in localised conduction slowing of a similar extent, with the degree of conduction slowing correlating positively with the patch size (Fig. 7B). The location of the patch was placed close to the site of latest activation time, such that differences in latest activation time correspond to the degree of conduction interruption in the patch. The mechanism by which each approach slows the local conduction velocity is most clearly illustrated with larger patch sizes in the largest tissue model (Fig. 7C) but is identical in the smaller model and smaller patch sizes (Fig. S7). Based on the experimental mapping results, the “APD-based” mechanism seems most likely to explain the observations; however, the simulations show that both mechanisms are viable. The “DAD-based” mechanism was more proarrhythmogenic through the potential development of re-entry, which did not occur in any of the APD-based simulations.

## 4 Discussion

In this work we utilise different electrical and optical mapping technologies to identify and explore possible mechanisms that could underlie conduction heterogeneity in the RyR2-R420Q preclinical model of CPVT. We found that local delays in activation occurred in R420Q hearts when beta-adrenergic signalling was increased along with pacing rate, despite overall ventricular activation time being similar to WT hearts. We identified spontaneous subthreshold DADs occurring in R420Q hearts that prolonged the APD of the beat immediately following the DAD, and delayed the activation of the subsequent beat. Computer modelling indicated that subthreshold DADs could explain local conduction slowing via an indirect mechanism involving I_Na_ inactivation during prolonged APs. Direct I_Na_ inactivation during DADs was less likely to explain our experimental results but was predicted to be more pro-arrhythmic and thus could have greater impact under more challenging conditions. Thus, DADs may be important for generating a substrate for re-entry by modulating both the refractory period and ventricular activation, in addition to their ability to trigger ectopic activity [33].

### 4.1 Increased susceptibility to rapid pacing induced ventricular arrhythmias

Exercise stress testing can reveal ventricular arrhythmias in CPVT patients at relatively low heart rate (∼110 bpm) that initially occur as single PVCs, whereas more intense exercise and further increased heart rate can provoke more severe bidirectional or polymorphic VT [34]. We found that rapid pacing in R420Q hearts predominantly induced single PVCs and couplets, whereas additional beta-adrenergic stimulation induced more severe longer-lasting runs of ventricular arrhythmias (Fig. 2D). Brief bursts of rapid pacing provoke arrhythmias more effectively than single premature stimuli in mouse hearts [21], which might be due to greater Ca^2+^ loading during repeated depolarizations. Geometric constraints in small mouse hearts make sustained VF less stable than in larger hearts, but conduction slowing near the centre of a rotor can facilitate re-entry [35] and such rotors have been described in CPVT mouse hearts [7,36].

### 4.2 Overall conduction velocity was not different in R420Q hearts

We did not find an overall difference in CV between R420Q and WT hearts under basal or stress conditions, which suggests that this is not a primary contributing factor in arrhythmias in this model. A recent optical mapping study employed dual Ca^2+^/V_m_ monitoring in heterozygous RyR2-R2474S CPVT hearts and similarly found no change in overall CV [36]. Slower CV was reported in atria and ventricles from homozygous mice with the RyR2-P2328S mutation [8,9], and was attributed to reduced Na_v_1.5 without a change in Cx43 expression [10], suggesting that perhaps CV slowing could contribute to arrhythmogenesis under more challenging conditions or mutations with more severe phenotypes. It is currently not known what, if any, role Na_v_1.5 or gap junctions play in the RyR2-R420Q phenotype. Dysfunction of pacemaker cells has also been implicated in CPVT, such as sinus bradycardia in RyR2-R420Q patients [13], and progressive or complete AV block [37,38]. In some cases we found progressive and variable PR intervals, resembling Wenckebach block, preceding VEB in CPVT hearts (e.g. Fig. 1C), although currently the mechanism responsible is not known. Arrhythmias were prevalent in R420Q hearts in sinus rhythm during perfusion with 1.8 mmol/L Ca^2+^, but further stimulation with ISO did not provoke additional arrhythmias (Fig. 1D). The increased heart rate during ISO stimulation may have reduced the occurrence of certain types of ventricular arrhythmia, similar to moderate tachypacing [29]. In contrast, the incidence of spontaneous arrhythmias was comparable between WT and R420Q hearts in 1 mmol/L Ca^2+^ but increased upon stimulation with ISO (Fig. S2), and thus the response to adrenergic stimulation may depend on the initial levels of Ca^2+^ signalling. CPVT patients will typically not exhibit arrhythmias under resting conditions but can develop tachycardias during physical or emotional stress [1].

### 4.3 Local conduction slowing as a substrate for re-entry

Dispersion of either ventricular activation or of AP repolarization can increase the heterogeneity of excitability throughout the heart and potential for re-entry [39]. We found increased activation dispersion during high physiological heart rate (∼10 Hz in mouse) combined with activation of beta-adrenergic signalling. Park *et al.* found rate- and catecholamine-dependent dispersion of wave propagation and the formation of re-entrant spiral waves in engineered human CPVT heart tissue [40]. The cause of conduction block was not specifically investigated but they did find an association with increased cytosolic Ca^2+^, whereas inhibition of CaMKII or ablation of the RyR2 serine 2814 phosphorylation site prevented catecholamine-stimulated conduction block and re-entry. Our data in adult murine myocardium are consistent with several of those findings and furthermore, we find that increased dispersion and/or block could result from the impact of subthreshold DADs on subsequent repolarization and inactivation of I_Na_.

Shortened diastolic interval during faster heart rate should increase Ca^2+^ loading and the likelihood of an AP upstroke coinciding with a DAD, which would cause direct I_Na_ inactivation and could induce local conduction block. This might occur more easily at shorter cycle lengths because of incomplete I_Na_ recovery. On the other hand, the time taken for SR refilling following a Ca^2+^ transient imparts an apparent refractory period on further spontaneous Ca^2+^ release, which would tend to suppress Ca^2+^ waves during faster pacing. The latency to first Ca^2+^ wave was shorter in isolated R420Q mouse ventricular myocytes from compared to WT, and shortened further still upon beta-adrenergic stimulation with a narrower latency distribution, which is expected to increase the likelihood of Ca^2+^ waves occurring before phase resetting occurs by arrival of the next AP [15]. Indeed, moderate atrial overdrive pacing reduced ventricular arrhythmias during catecholamine stimulation in CASQ2-null mice and reduced DADs in isolated ventricular myocytes [29].

### 4.4 Functional consequences of subthreshold DADs

Focal DADs in the myocardium can induce PVCs [33], but are normally unlikely to do so due to the large number of cells (∼800,000) that need to undergo depolarization fairly synchronously for triggered APs to propagate [3]. However in anatomically distinct areas, such as the His-Purkinje junction [41], border zone of myocardial infarction [4] or pulmonary vein sleeves [6], subthreshold DADs may induce action potentials in nearby cells with heterogeneous populations or with reduced electrotonic load. Afterdepolarizations in the His-Purkinje system may be an important trigger for arrhythmias in CPVT [7]. Cardiomyocyte-specific knockout of CASQ2 increased prevalence of VT, whereas chemical ablation of the endocardium reduced arrhythmia burden, and it was suggested that subthreshold DADs in the endocardium could induce retrograde activation of Purkinje fibres and subsequently PVCs [41].

Subthreshold DADs and delayed phase 3 repolarization have been suggested as possible causes of ECG U-waves [42]. Interestingly, augmented U-waves were reported in patients with the RyR2-R420Q mutation, particularly during repolarization of the first beat following a post-extrasystolic pause [12]. We observed subthreshold DADs in R420Q hearts but these would be unlikely to account for U-waves because they occurred towards the end of a diastolic pause. It is possible that APD lengthening caused by reduced CICR following a DAD during a prolonged pause might manifest as post-extrasystolic U-waves. In that situation, subthreshold DADs might increase repolarization variability, as was shown by Johnson et al. in canine hearts with impaired repolarization reserve during catecholamine stimulation [11]. The same study also found that small current injections in current clamped myocytes, sufficient to induce depolarizations of similar magnitude to DADs but without SR Ca^2+^ release, did not cause APD lengthening, indicating that reduced CICR following Ca^2+^ waves is critical. Some repolarization abnormalities have been reported in CPVT patients, such as increased short term QT variability that was irregular rather than alternan behaviour [43], but it is unclear how prevalent such abnormalities are in CPVT.

### 4.5 *In silico* modelling of DAD effects on activation in 2D tissue

Analyses were performed in which simulation data were matched to experimental data regarding the total area mapped and the inter-electrode spacing. However, at these small spatial scales, the conduction and APD heterogeneity in the models was significantly smaller than observed in tissue, making like-for-like comparisons of experimental and simulation data challenging. Therefore, the simulations are intended for mechanistic analysis only, and results presented are at a larger spatial scale than the mapping data. It is not surprising that the spatial heterogeneities in the computational model were smaller than observed in tissue at this scale, as computer models of traditional mechanisms of inter-cellular coupling, i.e., a discretisation of the mono- or bi-domain equations, have been known to fail to reproduce repolarisation heterogeneities over small spatial scales, that may be observed in mapping data or required for the mechanisms of dynamics such as discordant alternans [44], while under the same parameters that give the correct CV. That is to say, other mechanisms such as ephaptic coupling and gap junction dynamics [[44], [45], [46]] may be required to enable the coupling strength between adjacent myocytes to differ depending on whether it is during the excitation or repolarisation phases, which could match CV under the same parameters that also enable larger repolarisation heterogeneities over small spatial scales. The present model does not account for these more sophisticated components of coupling, and therefore this is likely the reason that we did not observe activation or repolarisation heterogeneities occurring to the same absolute degree over such a small spatial area as was mapped. Furthermore, the approximation of any discretised tissue model wherein a single node represents a collection of myocytes may also contribute to the inability to capture heterogeneities over such small spatial scales.

These simulations provide mechanistic tests rather than complete replication of the experimental conditions and their purpose was to explore whether the two independent mechanisms for DAD mediated conduction block (“direct I_Na_ inactivation” and “APD prolongation”) could explain the experimental observations, and if so, how they may differ in presentation. For instance, the upper panels in Fig. 7 demonstrate how the two mechanisms can work in principle and hence different model conditions were used, such as resting potential and pacing cycle length, to illustrate these mechanisms as clearly as possible. Altogether the simulation data do provide useful mechanistic insight to explain the mapping data, and this is supported by the ability to capture key features of the observed dynamics while also exploring different underlying causes.

## 5 Limitations

Currently, the only mammalian model of human CPVT mutations is mouse, but there are well-known limitations of using this species for cardiac excitation-contraction studies (see [47] for a review). The rodent heart rate is much faster than larger mammals, such as humans, and consequently the AP is shorter and more triangular due to different ion channel expression profile (e.g. greater reliance on I_to_ for repolarization, and smaller contribution of I_NCX_ versus SERCA2a for Ca^2+^ removal [48]). hiPSC-CM may be a possible alternative model for some studies. Indeed, Ca^2+^ handling abnormalities have been described in R420Q patient derived hiPSC [14,16] and in isogenic CRISPR knock-in hiPSC-CM carrying the R420Q mutation [17]. However currently even sophisticated CPVT engineered heart tissue does not fully replicate intact hearts (e.g. lacking in cardiac conduction system) [40], thus for this study it was necessary to use mouse. Fluorescent indicators and mechanical uncouplers used in optical mapping experiments can influence the excitability and electrical properties of the heart and parameters measured (e.g. [49,50] and see [51] for a recent review). While concentrations were kept to a minimum, there is a trade off against achieving sufficient signal to analyse subregions of the heart when signal averaging is not possible due to the stochastic nature of DADs.

## 6 Conclusion

We show that increased activation dispersion in the ventricle of intact R420Q mouse hearts could be caused by subthreshold DADs that prolong the APD in affected regions and indirectly delay the activation of subsequent APs by increasing I_Na_ inactivation during the AP. Targeting dysfunctional Ca^2+^ handling or Ca^2+^ regulatory mechanisms could have therapeutic potential to rectify some of these arrhythmogenic consequences in CPVT. For instance suppressing CaMKII activity reduced dispersion of activation in engineered human CPVT heart tissue [40], and inhibiting RyR2 with dantrolene reduced dispersion of repolarization in a guinea pig model of non-ischaemic heart disease [52]. The mapping technologies employed here could provide valuable mechanistic insight into how existing or novel therapies modulate arrhythmias.

## CRediT authorship contribution statement

**Ewan D. Fowler:** Writing – review & editing, Writing – original draft, Visualization, Resources, Project administration, Methodology, Investigation, Funding acquisition, Formal analysis, Conceptualization. **Salimata L. Diakite:** Investigation, Formal analysis. **Ana M. Gomez:** Writing – review & editing, Supervision, Resources, Investigation, Funding acquisition, Formal analysis. **Michael A. Colman:** Writing – review & editing, Writing – original draft, Visualization, Software, Investigation, Funding acquisition, Formal analysis.

## Declaration of Generative AI and AI-assisted technologies in the writing process

AI-assisted technology was not used in the preparation of this work.

## Sources of funding

This research was supported by a 10.13039/501100000274British Heart Foundation Intermediate Basic Science Research Fellowship to EDF (FS-IBSRF-21-25071), 10.13039/501100001665Agence Nationale de la Recherche (ANR-19-CE14-0031-01 & ANR-23-CE14-0009-02) and 10.13039/100000002NIH (2R01HL055438-22) to AMG, and 10.13039/501100000265Medical Research Council Career Development Award (Grant Number MR/V010050/1) to MAC.

## Declaration of competing interest

The authors declare the following financial interests/personal relationships which may be considered as potential competing interests: Ewan Fowler reports financial support was provided by British Heart Foundation. If there are other authors, they declare that they have no known competing financial interests or personal relationships that could have appeared to influence the work reported in this paper.

## Appendix A Supplementary data

Supplementary material Image 1
