# Supplementary material for: Disruption of ventricular activation by subthreshold delayed afterdepolarizations in RyR2-R420Q catecholaminergic polymorphic ventricular tachycardia
Source: J Mol Cell Cardiol Plus. 2025 Jun 11;13:100466. doi: 10.1016/j.jmccpl.2025.100466 (PMC12221671; doi:10.1016/j.jmccpl.2025.100466)
Supplement: Supplementary file 1 — Supplementary material [file mmc1.docx]

**Supplemental Figures and Figure Legends**

**
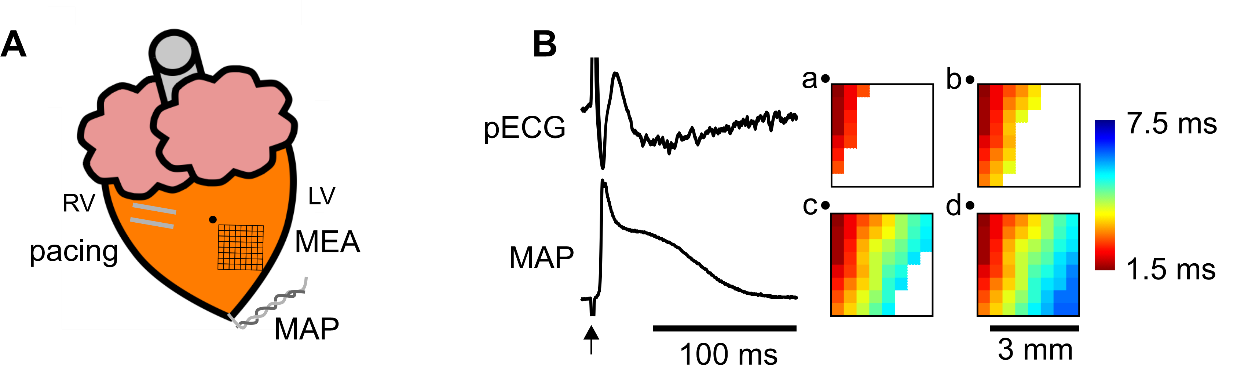
**

**Supplementary Figure 1.** Multielectrode array (MEA), monophasic action potential (MAP) electrode, and stimulating electrode placement during perfused heart experiments. **A** Cartoon diagram illustrating the position of recording and stimulating electrodes during MAP and MEA experiments. MAP electrodes were placed on the LV apex. Stimulating electrodes were placed on the RV free wall. MEA was positioned on the LV free wall, with electrode #1 (indicated by a black dot) oriented towards the pacing site. **B** Exemplar pseudo-ECG (pECG), MAP and corresponding activation maps recorded in response to an electrical stimulus delivered at the time indicated by a black arrow. The earliest activation occurs near electrode #1 (indicated by a black dot), then the electrical wave propagates towards the bottom right corner.


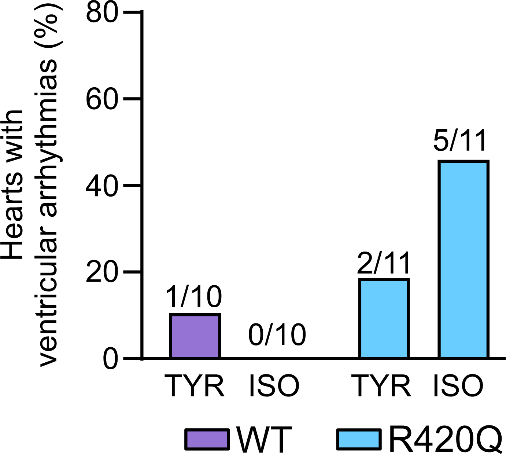


**Supplementary Figure 2.** Incidence of spontaneous ventricular arrhythmias recorded during sinus rhythm in Langendorff-perfused WT and R420Q hearts during perfusion with 1 mmol/L Ca^2+^ Tyrode’s (TYR) before and after 100 nmol/L isoproterenol (ISO). N = 10 WT and 11 R420Q hearts.

**
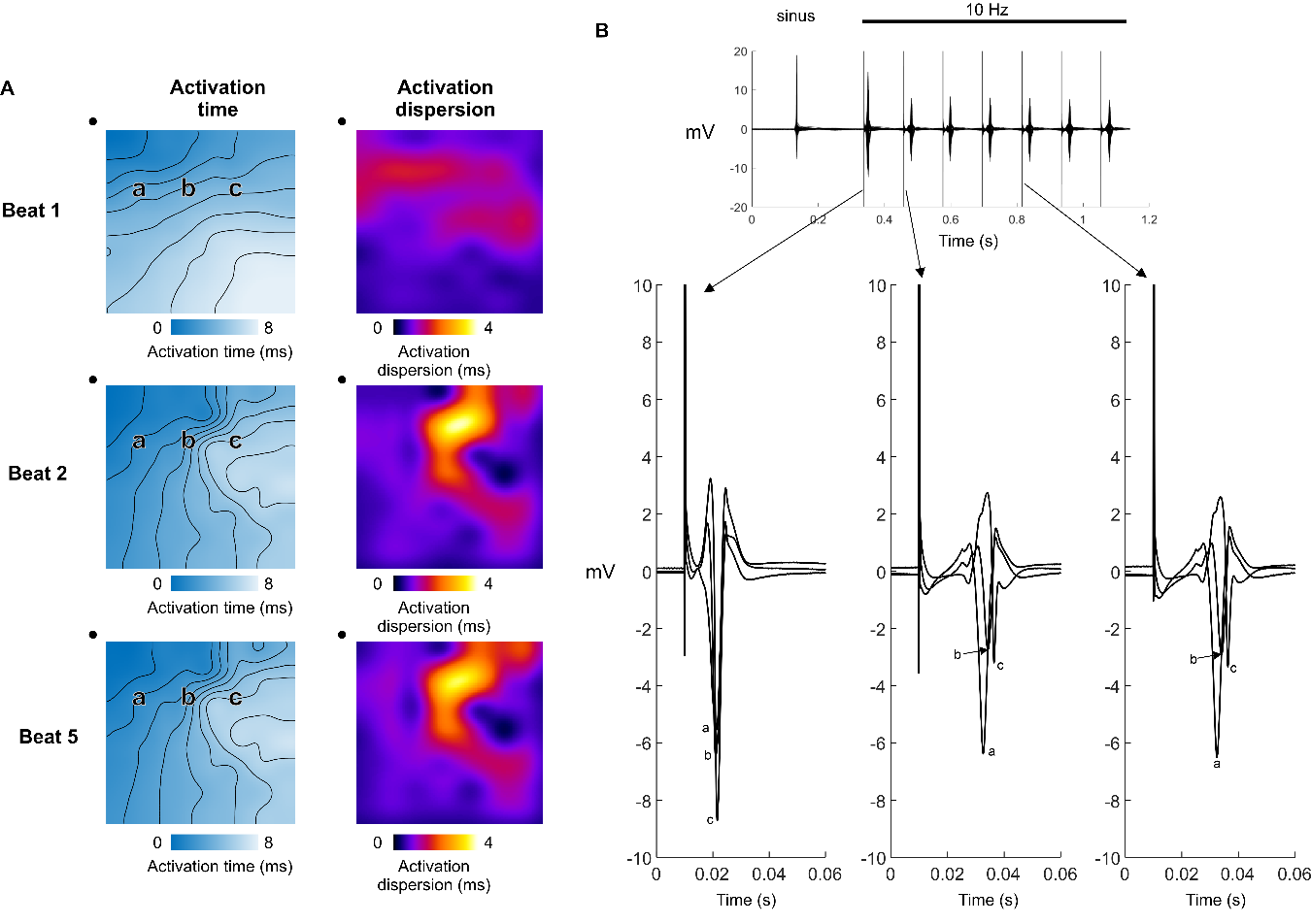
**

**Supplementary Figure 3.** Electrograms recorded in the region of delayed activation from Fig. 4. **A** Activation maps during the first, second and fifth electrically paced beat at 10 Hz. **B** (top panel) Combined electrogram showing overlay of all 64 MEA channels. (Lower panels) Individual MEA channels spanning the region of conduction block (indicated by labels ‘a’, ‘b’, and ‘c’ in panel A).


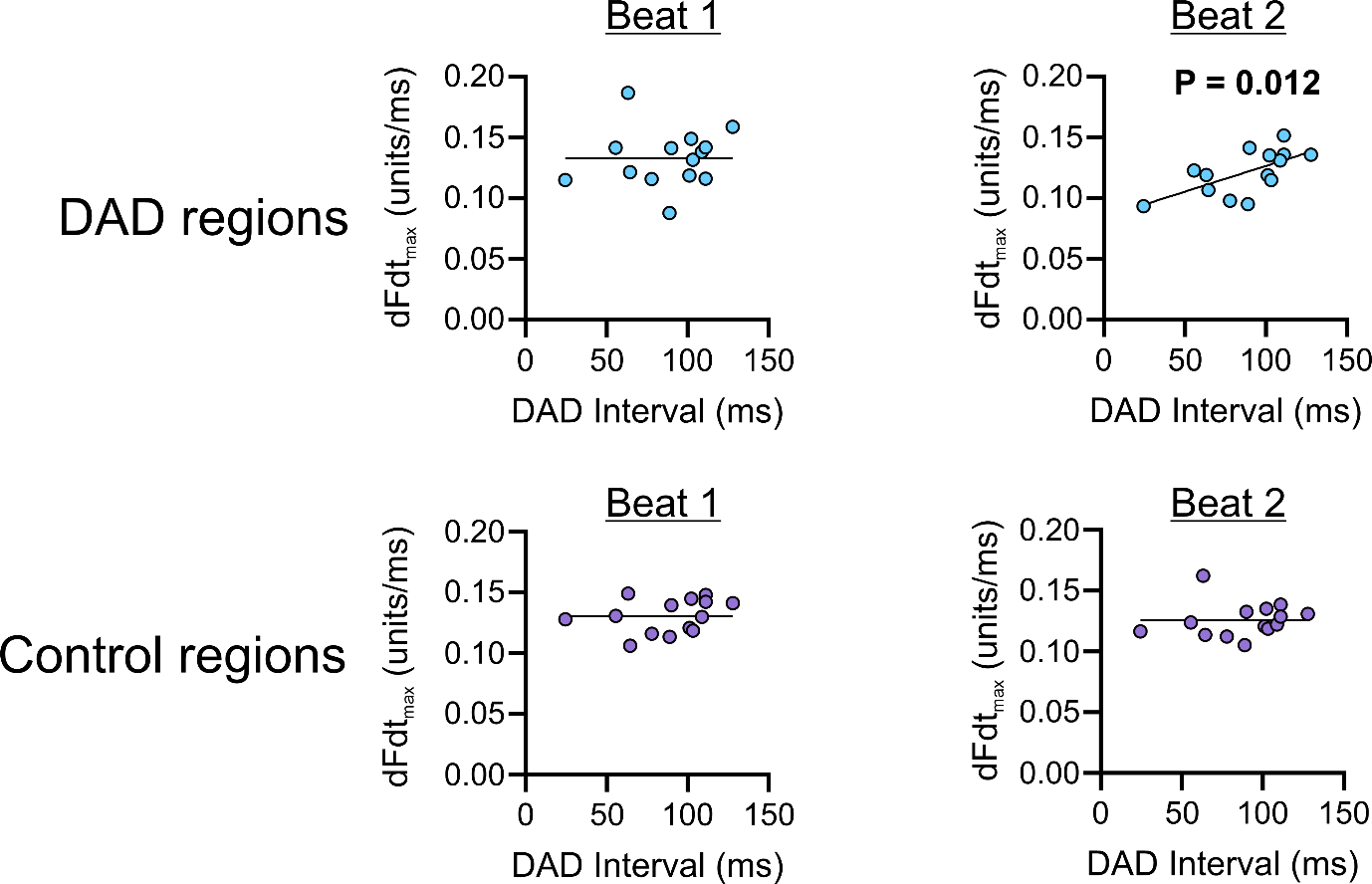


**Supplementary Figure 4.** Optical AP upstroke maximum rate of rise (dFdt_max_) plotted as a function of the preceding DAD interval. Data points represent the dFdt_max_ of the first (left panels) or second AP (right panels) following a DAD in regions where the DAD occurred (blue symbols) or in corresponding control regions remote to the DAD (purple symbols). DADs occurring within 150 ms of an AP were analysed and over this range there was a fairly linear relationship between DAD interval and dFdt_max_ of the second beat in DAD regions that was significant (P = 0.012), but not in beat 1 (P = 0.71) or in control regions during either beat 1 (P = 0.33) or beat 2 (P = 0.68). 14 events from N=9 R420Q mice were analysed.


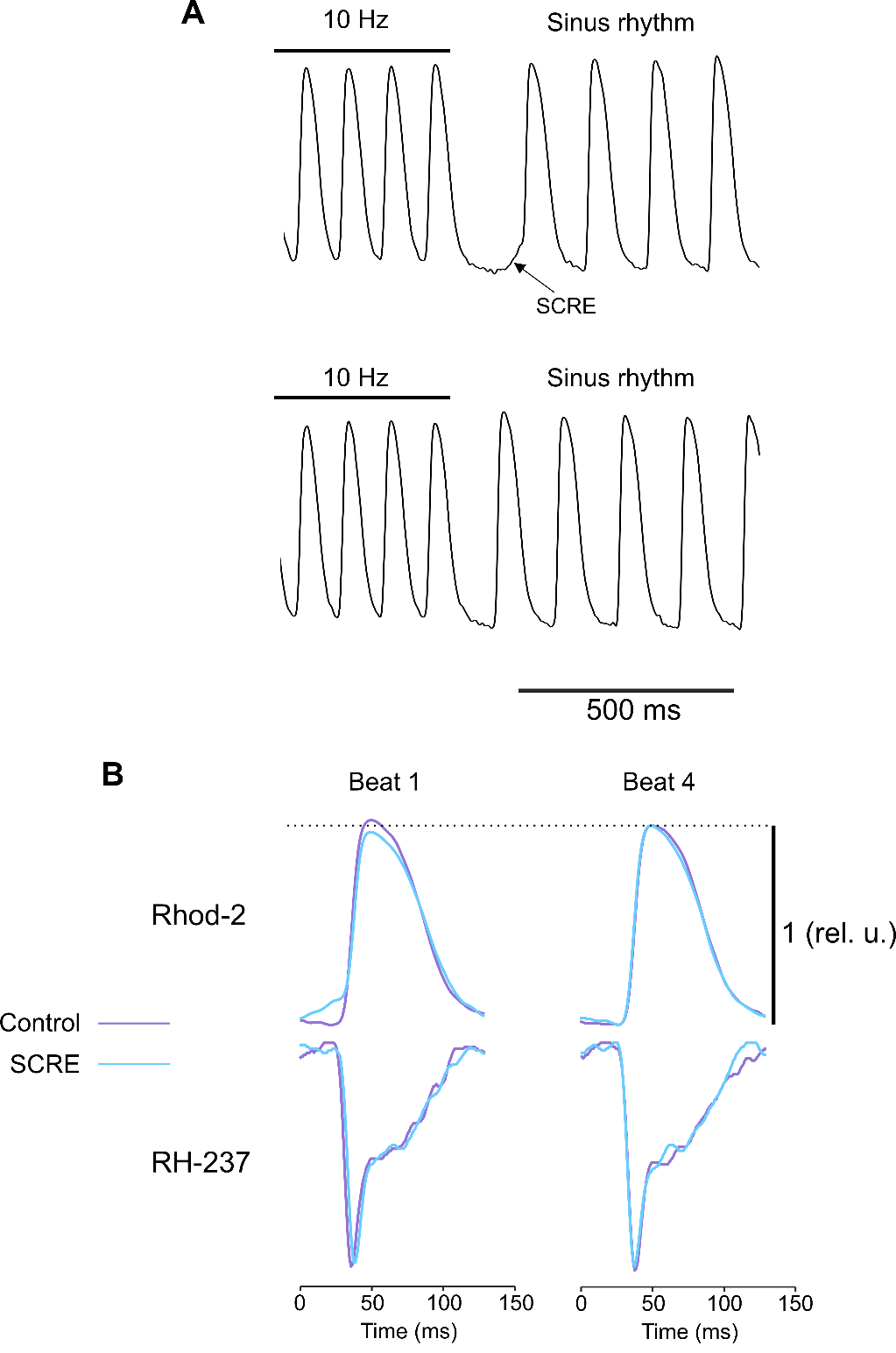


**Supplementary Figure 5.** Simultaneous Ca^2+^ and voltage optical mapping of spontaneous Ca^2+^ release events (SCRE) in an R420Q mouse heart during stimulation with 1.8 mM external [Ca^2+^] and 1 µmol/L ISO. **A** (*Top panel*) Average intensity plots of Rhod-2 signal in a small region of the ventricle (500 x 500 µm) during pacing at 10 Hz followed by cessation of pacing and return of sinus rhythm. An SCRE occurs just prior to the first sinus beat. (*Lower panel*) The stimulation protocol was repeated ~13 s later, but on this occasion no SCRE occurred. The recording site was identical in the top and bottom panels. **B** Aligned overlay comparing the Rhod-2 (top) and RH-237 signals (bottom) during the first (left panels) and 4^th^ (right panels) sinus beat during the recording with (blue lines) and without SCRE (purple lines). An SCRE precedes the start of phase 0 of the AP. The first Ca^2+^ transient amplitude is slightly reduced and the duration slightly increased following the SCRE. Rhod-2 signals are normalised to the 4^th^ beat of each recording (rel. u. = relative units).


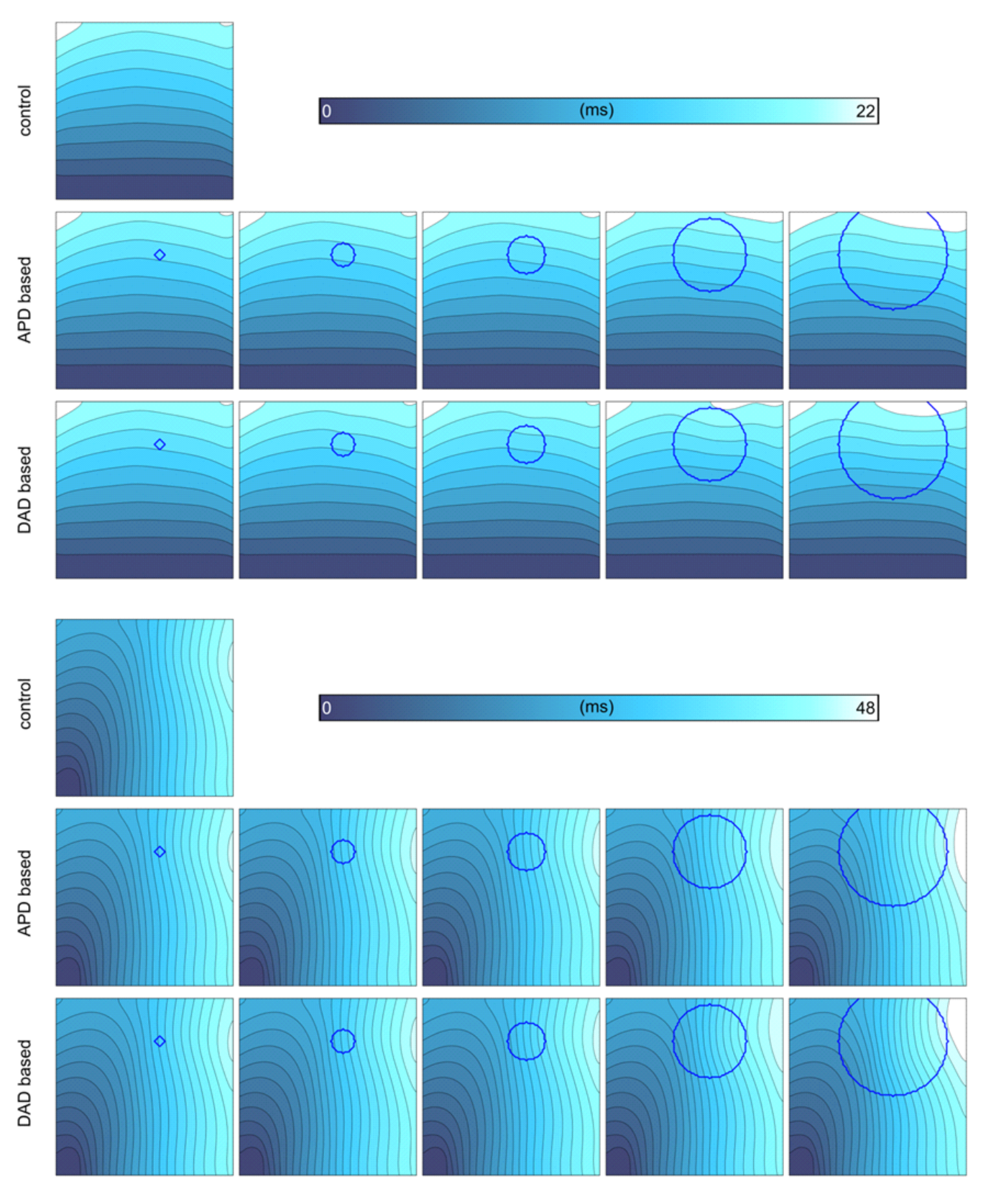


**Supplementary Figure 6.** Activation maps with different patch sizes and pacing locations in the 100x100 tissue model, implementing the two different conduction block mechanisms. The top panels correspond to a stimulus applied along the bottom edge; the lower panels to a stimulus applied in the lower left corner. Patch sizes shown correspond to diameters of 2, 6, 10, 20 and 30 nodes. The SCRE (spontaneous release function) parameters selected from this approach for analysis and visualisation were: median duration = 100 ms, duration distribution width (67% of population) = 50 ms, mode of latency time distribution = 70 ms relative to the previous AP upstroke (note a pacing frequency of 10 Hz), left and right widths of the skewed latency time distribution = 50 and 80 ms, respectively. The observed results were not specific to this parameter combination, and varied smoothly between no effect through conduction block to full triggered focal excitation.


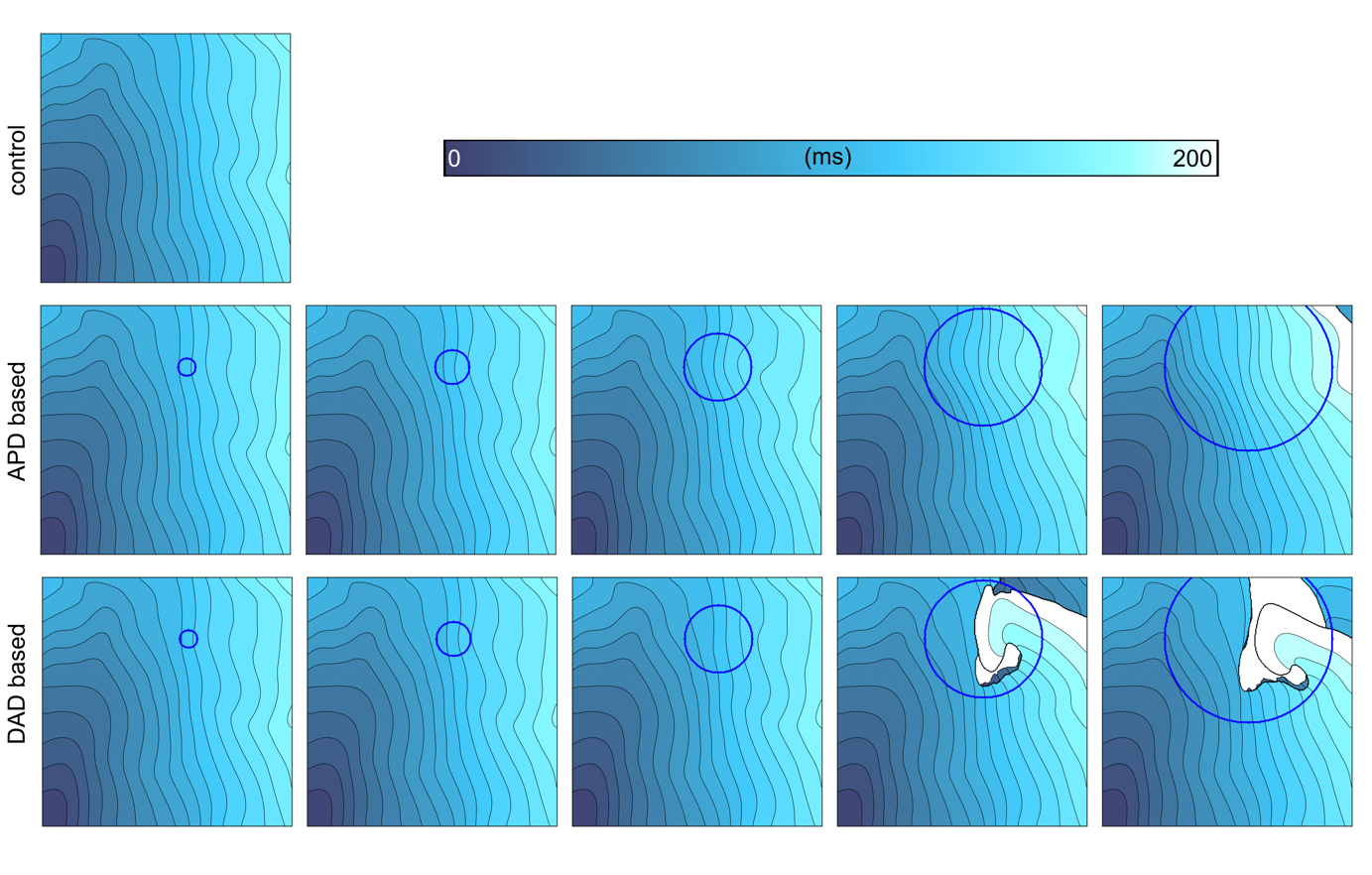


**Supplementary Figure 7.** Activation maps with different patch sizes in the 300x300 tissue model, implementing the two different conduction block mechanisms. Patch sizes shown correspond to diameters of 10, 20, 40, 70 and 100 nodes.


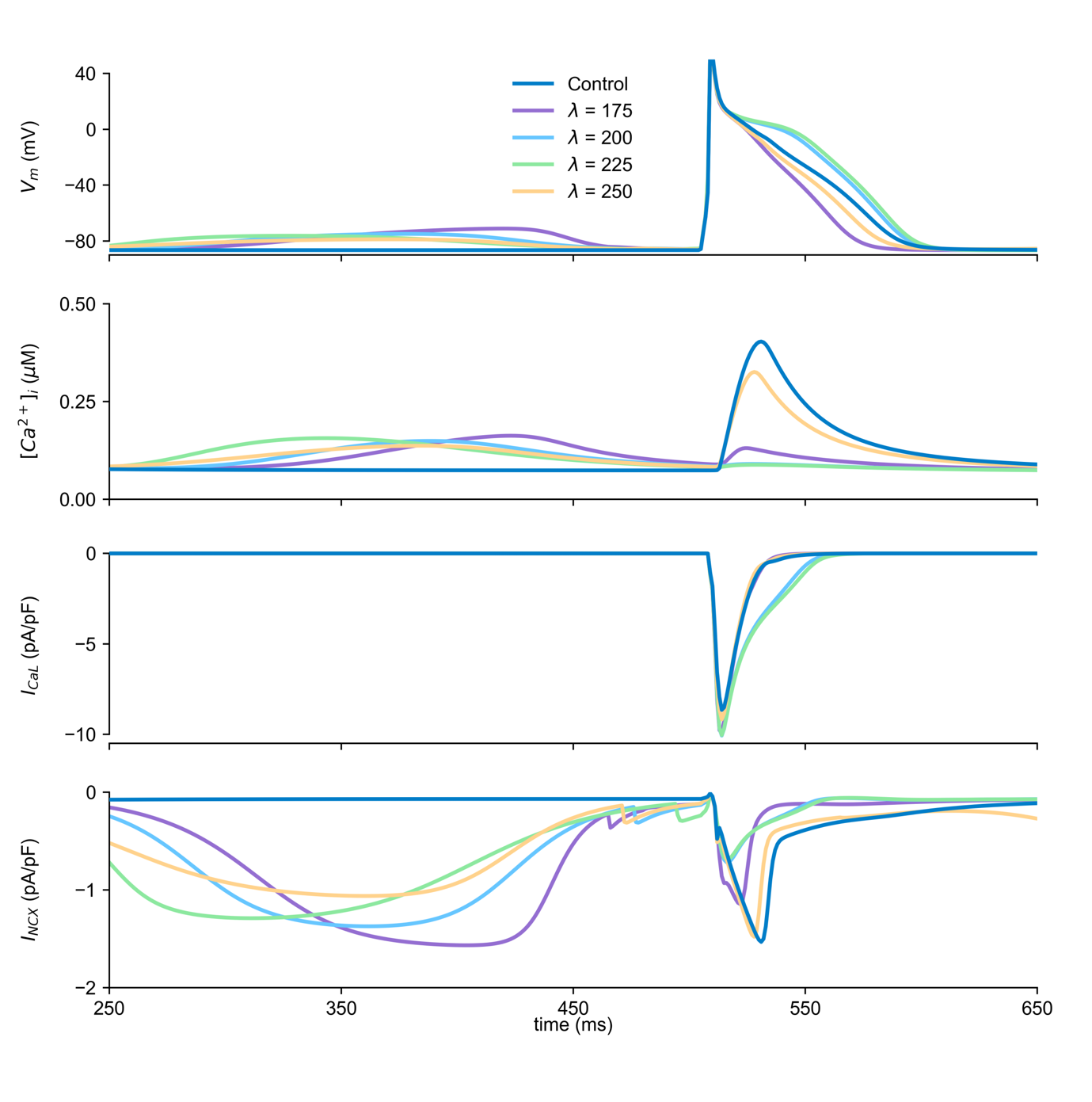


**Supplementary Figure 8.** Relationship between DAD properties and subsequent AP properties. Traces are shown for the voltage (upper), calcium transient (second), *I*_CaL_ (third) and *I*_NCX_ (fourth) for simulations with different durations (λ, ms) of spontaneous release function which underpin the DAD. Both APD prolongation and APD shortening are observed, dependent on the magnitude of the calcium transient induced by CICR. The essentially binary impact on *I*_CaL_, where it either undergoes calcium-induced inactivation or does not, which is a model-dependent feature, is clear.

**Supplementary Tables**

**
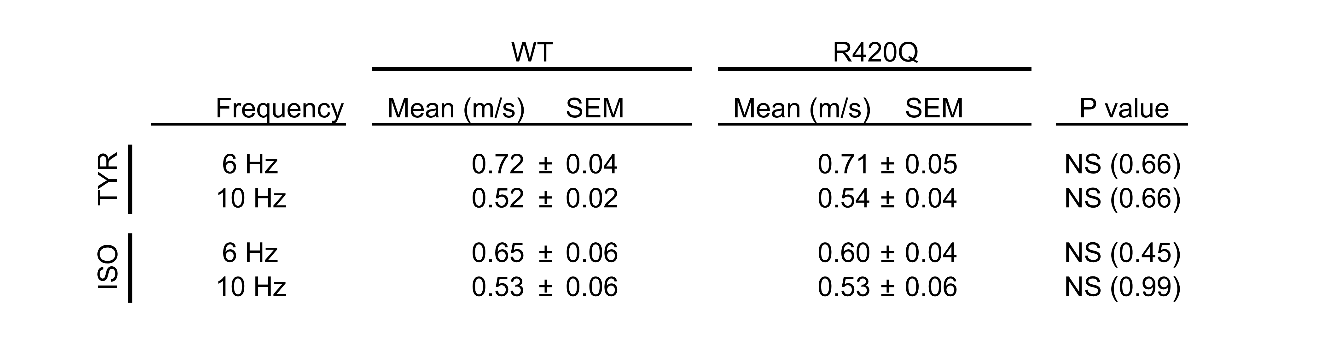
**

**Supplementary Table 1.** Conduction velocity calculated from local conduction vectors during electrical pacing at 6 or 10 Hz, in 1 mmol/L TYR before and after 100 nmol/L ISO. Data were analysed using the same recordings as Fig. 3.
